# 2263 Creating a reference analytics morphomics population from surgical patient cross-sectional imaging

**DOI:** 10.1017/cts.2018.280

**Published:** 2018-11-21

**Authors:** Katherine He, Brian Derstine, Sven Holcombe, Nicholas C. Wang, Stewart C. Wang

**Affiliations:** 1 University of Michigan School of Medicine, Ann Arbor, MI, USA; 2 Department of Surgery, Morphomics Analysis Group, University of Michigan, Ann Arbor, MI, USA

## Abstract

OBJECTIVES/SPECIFIC AIMS: Patient factors such as body mass index and functional status are commonly used in surgical decision-making and prediction of outcomes. Morphomic analysis uses semi-automated 3D cross-sectional imaging analysis to quantify tissue, organ, and bone geometry and density. These data can be used to assess patient health status. There is an emerging trend of using morphomic variables such as muscle mass and bone mineral density to predict surgical and medical outcomes. In certain cases, it has been shown to predict cancer survival more accurately than conventional staging methods. With the growing popularity of morphomic analysis, it is vital to establish baseline variability against which patient populations can be validated. Of populations receiving radiographic imaging, trauma patients are approximately representative of the general population. We created a reference population of morphomic variables from over 6000 University of Michigan patients presenting with trauma. METHODS/STUDY POPULATION: Computed tomography (CT) scans were obtained for all patients who underwent scans for trauma indications at the University of Michigan starting from April 1998. High throughput image processing algorithms written in MATLAB 2015a were used to semi-automatically process chest, abdomen, and pelvis CT scans. Scans were referenced to a common coordinate system based on vertebral levels and body anatomy. Measurements of adiposity, muscle group, and bone density measurements were performed at each level. Percentile curves of morphomic measures of body composition by age and sex were created. The reference population dataset is periodically updated and is publicly accessible. RESULTS/ANTICIPATED RESULTS: As of July 2017, over 6000 patients aged 1–81 years have been included in the Reference Analytics Morphomics Population. Patient CT scans were analyzed at the T10, T11, T12, L1, L2, L3, and L4 vertebral levels. Morphomic measures analyzed include body depth, body cross-sectional area, vertebral trabecular bone density, visceral fat area, fascia area, subcutaneous fat area, central back fat, and psoas muscle area. DISCUSSION/SIGNIFICANCE OF IMPACT: We created reference curves for several morphomic variables from a Reference Analytic Morphomics Population of over 6000 University of Michigan patients presenting with trauma.

